# Repeated 6-Hz Corneal Stimulation Progressively Increases FosB/ΔFosB Levels in the Lateral Amygdala and Induces Seizure Generalization to the Hippocampus

**DOI:** 10.1371/journal.pone.0141221

**Published:** 2015-11-10

**Authors:** Carmela Giordano, Jonathan Vinet, Giulia Curia, Giuseppe Biagini

**Affiliations:** 1 Department of Biomedical, Metabolic and Neural Sciences, Laboratory of Experimental Epileptology, University of Modena and Reggio Emilia, Modena, Italy; 2 Department of Neurosciences, NOCSAE Hospital, AUSL Modena, Italy; Georgia Institute of Technology, UNITED STATES

## Abstract

Exposure to repetitive seizures is known to promote convulsions which depend on specific patterns of network activity. We aimed at evaluating the changes in seizure phenotype and neuronal network activation caused by a modified 6-Hz corneal stimulation model of psychomotor seizures. Mice received up to 4 sessions of 6-Hz corneal stimulation with fixed current amplitude of 32 mA and inter-stimulation interval of 72 h. Video-electroencephalography showed that evoked seizures were characterized by a motor component and a non-motor component. Seizures always appeared in frontal cortex, but only at the fourth stimulation they involved the hippocampus, suggesting the establishment of an epileptogenic process. Duration of seizure non-motor component progressively decreased after the second session, whereas convulsive seizures remained unchanged. In addition, a more severe seizure phenotype, consisting of tonic-clonic generalized convulsions, was predominant after the second session. Immunohistochemistry and double immunofluorescence experiments revealed a significant increase in neuronal activity occurring in the lateral amygdala after the fourth session, most likely due to activity of principal cells. These findings indicate a predominant role of amygdala in promoting progressively more severe convulsions as well as the late recruitment of the hippocampus in the seizure spread. We propose that the repeated 6-Hz corneal stimulation model may be used to investigate some mechanisms of epileptogenesis and to test putative antiepileptogenic drugs.

## Introduction

The 6-Hz corneal stimulation model was introduced in early fifties [1] to screen new anticonvulsants [2]. This model has been recently rediscovered to test alternative approaches for drug-resistant seizures [3]. Accordingly the ketogenic diet, which is known to be a valid treatment for refractory seizures [4,5], was found to be effective in counteracting seizures induced by the 6-Hz corneal stimulation [3]. When compared with other models based on electrical stimulation, the 6-Hz paradigm is characterized by the induction of minimally convulsive or non-convulsive seizures with automatized behaviors, defined as “psychomotor seizures” [1,3,6]. Rarely animals develop generalized tonic-clonic seizures with the 6-Hz protocol, whereas tonic seizures are predictably obtained in other models based on electrical stimulation, such as the maximal electroshock seizure model [7].

Few studies addressed the involvement of brain regions in psychomotor seizures observed in the 6-Hz model [8,9]. Analysis of c-Fos immunoreactivity 2 h after 6-Hz corneal stimulation revealed a pattern of wide expression, with highest levels of immunoreactivity in the piriform cortex (PIR), and moderate expression in neocortex, cingulate and perirhinal (PER) cortices, and amygdala [8]. A further study based on the autoradiographic analysis of ^14^C-2-deoxyglucose uptake described enhanced activity in neocortex, lateral amygdala (LA), and caudate-putamen (CPu) few min after 6-Hz corneal stimulation [9], partially confirming the results obtained by mapping c-Fos immunoreactivity. Although the hippocampus has been frequently involved in refractory seizures, as in the case of patients affected by temporal lobe epilepsy (TLE), evidence for the involvement of hippocampal structures is currently lacking in the 6-Hz corneal stimulation model [8,9], which suggests that this approach could present important limitations.

In the tentative to reconsider the role of the hippocampal formation and related limbic regions in the 6-Hz model, we modified the stimulation protocol in order to simulate the occurrence of recurrent seizures, as it is usually observed in patients. In addition, to map neuronal activation, we used an antibody against FosB/ΔFosB-related antigens, which were previously characterized as reliable markers in different models of epilepsy [10–15]. By administering the 6-Hz stimulation protocol up to four sessions, we observed a rapid aggravation of convulsions and a shortening of non-convulsive behaviors which preceded the recovery to normality. These changes were associated with a marked variation in the pattern of FosB/ΔFosB expression and with the spread of epileptiform electrographic activity to the hippocampus.

## Materials and Methods

### Animals

A total of forty-four male (six week-old) CD-1 mice (Charles River, Calco, Italy) were used in this study. They were housed in a specific pathogen-free facility under controlled environment with *ad libitum* access to water and food. Of these, 8 mice were stimulated once, 8 were stimulated twice, 8 were stimulated thrice, and 8 were stimulated four times. Six mice receiving a sham corneal stimulation were used as control group. One day after the last stimulus session, mice were perfused and their brains were collected for immunohistochemistry experiments described below. Data of first stimulation were obtained from all animals included in the experiment. So, the number of used animals was 32 for 1 stimulus session, 24 for 2 stimulus sessions, 16 for 3 stimulus sessions, and 8 for 4 stimulus sessions. Only for data aimed at elucidating changes occurred over repeated stimulations (specified in the text), data obtained from the 8 animals stimulated 4 times were used in order to compare the repeated measures.

Additionally, 6 mice were used to obtain video-electroencephalographic (EEG) recordings and underwent 4 stimulus sessions. Their brains were not used for immunohistochemistry experiments. All the procedures were approved by the Italian Ministry of Health and performed in accordance with the European Directive 2010/63/EU.

### Corneal stimulation protocol

Mice were stimulated once daily and let to recover for 2 days before being stimulated again. Corneal stimulation was as described by others [1,8,9]. Briefly, ocular anesthetic (0.4% oxybuprocaine hydrochloride eye drops, Novesin, Novartis, Switzerland) was applied 10 min before stimulation. Stimulation (fixed current intensity of 32 mA, pulse width of 0.2 ms, duration of 3 s, frequency of 6 Hz) was delivered via corneal electrodes connected to a stimulator (ECT Unit 5780; Ugo Basile, Comerio, Italy). Corneal electrodes were wetted with 0.9% saline immediately before the test to enhance the conductivity of the current. During stimulation, mice were manually restrained and released immediately after the current application to monitor the duration of seizures and to score the type of convulsions, with the support of video recordings. Seizure severity was ranked according to the following score: 1, stunned posture and eye blinking; 2, head nodding, Straub tail, and repetitive rhythmic movements (stereotypies), such as chewing; 3, unilateral or alternating forelimb clonus; 4, generalized tonic-clonic convulsions without loss of posture, or rearings; 5, generalized tonic-clonic convulsions with loss of posture. Seizures were first scored immediately after corneal stimulation by direct observation, then reanalyzed on video recordings to quantify the duration of behavioral changes by an investigator unaware of the stimulation session.

### Electrodes implantation for EEG recordings

For electrode implantation, mice were anesthetized with ketamine-xylazine (150–10 μg/g). After deep anesthesia was reached (assessed with deep breath, loss of tail and eye reflexes), skin was shaved, disinfected with povidone-iodine 10% (Betadine^®^ skin solution; Meda Pharma, Milano, Italy), and cut open to expose the scalp. Guiding holes were drilled and epidural electrodes (stainless steel Ø = 1 mm; PlasticsOne, Roanoke, VA, USA) were implanted in frontal (bregma 0 mm, 3 mm lateral from midline) and occipital cortices (bregma -3.5 mm, 3 mm lateral from midline) of right hemisphere. One electrode was implanted below lambda on the midline and used as reference. In addition, a hole was performed on the left hemisphere to allow implantation of one intracerebral bipolar electrode (made of two wires twisted, final Ø = 0.15 mm) in the Cornu Ammonis (CA)1 hippocampal region (bregma -2.5 mm, 2 mm lateral from midline, 1.1 mm deeper than cerebral surface). Electrodes were connected by steel wire to terminal gold pins (Bilaney Consultant GmbH, Düsseldorf, Germany) that were inserted in a plastic pedestal (PlasticsOne) cemented on the mouse head. At the end of the surgery, gel containing 2.5 g lidocaine chloride, 0.5 g neomycin sulfate and 0.025 g fluocinolone acetonide (Neuflan^®^ gel; Molteni Farmaceutici, Scandicci, FI, Italy) was applied to reduce pain and risk of infection. Mice were kept under heat lamp and monitored until complete recovery from anesthesia; then they were housed in single cages without grids or environmental enrichments to reduce risk of headset lost.

### Video-EEG recordings

Mice were placed in cages without cover to allow cable connection between headset and preamplifiers. Electrical brain activity was filtered (0.3 Hz high-pass, 500 Hz low-pass), acquired at 1 kHz per channel, and stored on personal computer as the mathematical subtraction of traces of recording electrodes minus traces of reference electrode (only for epidural electrodes), using a PowerLab8/30 amplifier connected to 4 BioAmp preamplifiers (ADInstruments; Dunedin, Otago, New Zealand). Videos were digitally captured by a camera connected to the computer and synchronized to the EEG traces by LabChart 7 Pro internal trigger.

### EEG analysis

EEG traces were offline digitally filtered (band-pass: high 50 Hz, low 1 Hz) and manually analyzed using LabChart 7 Pro software (AD Instruments) by expert raters. Seizures were characterized by epileptiform EEG patterns occurring right after stimulus artifact.

For each seizure, the power spectrum was obtained by fast Fourier transformation of the EEG waveforms (LabChart 7 PRO, ADInstruments), and used to obtain the mean power spectra for the experimental group. For each mouse, the maximum value of power was considered as the peak value. Peak values of power spectra of baseline, ictal and post-ictal depression recorded from frontal electrode were compared to investigate whether changes had occurred in the different states. Peak values of power spectra of ictals, recorded from frontal, occipital and intracerebral electrodes, were compared to investigate whether seizure changes had occurred from first to last stimulus session.

### Immunohistochemistry

Mice were deeply anesthetized with isoflurane (~10 s), perfused transcardially with phosphate buffered saline (PBS, pH 7.4), followed by Zamboni’s fixative (pH 6.9). Brains were removed and postfixed at 4°C in the same fixative [14] for 24 h. Tissues were transferred to 15% and 30% sucrose solution and horizontal sections (50 *μ*m) were subsequently cut using a freezing-sliding microtome (Leica SM2000 R; Leica, Nussloch, Germany).

Sections were washed three times in PBS, treated with 3% H_2_O_2_ in PBS (10 min), and blocked 1 h in PBS containing 5% normal goat serum and 0.1% Triton X-100 (Tx). Then sections were respectively incubated overnight (O/N) at 4°C with rabbit polyclonal anti-FosB/ΔFosB (H-75, sc-7203, Santa Cruz Biotechnology, CA, USA; 1:250) or rabbit polyclonal anti-Iba1 (019–19741, Wako Pure Chemicals; 1:1000). The next day, sections were incubated 1 h with a biotinylated anti-rabbit secondary antibody (Vector Laboratories, Burlingame, CA, USA; 1:200). After additional washing, sections were incubated 1 h with the avidin-biotin-peroxidase complex (Elite ABC Kit; Vector Laboratories). The immunostaining was developed in 0.05% 3,3-diaminobenzidine tetrahydrochloride for 5 min (Sigma-Aldrich, Milan, Italy) by adding 0.03% H_2_O_2_.

### Image Analysis

Immunostained sections were evaluated with an Axioskop microscope (Carl Zeiss Vision GmbH, Munchen, Germany) equipped with a 10X objective and for each area of interest, images were digitally captured by a Sony CCD-IRIS B–W video camera. The number of immunoreactive cells per mm^2^ was quantified and analyzed using the image analysis software KS300 (Carl Zeiss Vision GmbH), as previously detailed [14,16]. All measurements were taken along the ventrodorsal direction of the brain (approximately 5–6 serial horizontal sections separated by 0.5 mm). A mouse brain atlas-C57BL/6J horizontal (http://www.mbl.org/atlas232/atlas232_frame.html) was used to identify brain sections relative to bregma from −8.04 to −5.04 mm for hippocampal CA1, CA3 and dentate gyrus (DG) regions, the subiculum (Sub), entorhinal cortex (EC) and PER. For the CPu, we included sections ranging from -6.24 to -3.84 mm relative to bregma. For LA and PIR we considered sections relative to bregma from -8.64 to -7.44 mm (approximately 1–2 sections for each brain). The border of all analyzed regions was manually traced and area of immunoreactivity, expressed in mm^2^, was automatically determined. Background values in sections were obtained from areas that did not contain any stained cell, such as the angular bundle. Cell counts were determined in each area as the number of immunopositive cells after transformation in D-circles (i.e., the diameter of circles having the same area as measured) by considering a minimum cut-off value of 7 μm. Cell counts were then divided by the sampled area and expressed as cellular density (n/mm^2^). All measurements were taken bilaterally and the final values represent the left-right average.

### Double immunofluorescence

Sections were rinsed and blocked 1 h in PBS containing 5% normal goat serum and 0.1% Tx in case of calcium/calmodulin-dependent protein kinase II (CaMKII) staining, or 0.02% Tx in the case of glutamic acid decarboxylase 67 (GAD67) staining. Sections were then incubated O/N at 4°C with rabbit anti-FosB/ΔFosB primary antibody (H-75, sc-7203, Santa Cruz Biotechnology; 1:250) and either mouse anti-CaMKII primary antibody (MA1–048; Pierce Biotechnology; 1:100) or mouse anti-GAD67 primary antibody (MAB5406; Chemicon International; 1:200). After washing, sections were incubated for 3 h with secondary antibodies Alexa Fluor 488-labeled goat anti-rabbit (A-11001; Invitrogen, Carlsbad, CA, USA; 1:500) and Alexa Fluor 594-labeled goat anti-mouse antibody (A-11005; Invitrogen, Carlsbad, CA, USA; 1:500). Sections were mounted and coverslipped with Mowiol. All fluorescent images were acquired using a Leica SP2 AOBS laser scanning confocal microscope (Leica Microsystems, Milan, Italy). Images for colocalization were captured at 40X magnification and the number of double-labeled cells were counted using Fiji software (http://fiji.sc/Fiji).

### Fluoro-Jade B

Brain sections were washed in PBS, mounted on gelatin-coated slides and dried at room temperature. Slides were immersed for 5 min into a solution containing 1% sodium hydroxide in 80% ethanol. Slides were then washed for 2 min in 70% ethanol followed by 2 min in distilled water, before being oxidized in 0.06% potassium permanganate for 15 min. Sections were then stained for 15 min in a 0.004% solution of Fluoro-Jade B (Millipore, Temecula, CA) diluted in 0.1% acetic acid. Slides were rinsed in deionized water for 3 min, dried on a pre-warmed hot plate at 50°C, then cleared in xylene and cover-slipped with Eukitt (EMS Inc, Hatfield, PA, USA) [17]. Images were acquired using a Leica SP2 AOBS laser scanning confocal microscope (Leica Microsystems, Milan, Italy). Horizontal brain sections from rats sacrificed 7 days after pilocarpine-induce *status epilepticus* were used as positive controls [18].

### Statistics

Peak values of power spectra obtained from different states (baseline, ictal, post-ictal depression and recovery) detected by the epidural electrode implanted in frontal cortex were compared using the Mann-Whitney rank sum test. The Chi-square test was used to compare the percentage of animals experiencing seizures with loss of posture, in the various groups of treatment. Time spent in total seizure activity was analyzed by Kruskal-Wallis one-way analysis of variance (ANOVA) on ranks, followed by Dunn's method for post-hoc comparisons. Time spent in convulsive or non-convulsive seizure components was analyzed by Friedman repeated measures ANOVA on ranks, followed by Tukey’s post-hoc test, and only animals receiving all 4 stimulus sessions were used for this analysis. Peak values of power spectra obtained from the four stimulus sessions for ictal events detected by the epidural and depth electrodes were compared using the Friedman repeated measures ANOVA on ranks test, followed by Tukey’s test. FosB/ΔFosB positivity values (both in immunohistochemistry and immunofluorescence experiments) were compared using one-way ANOVA followed by Tukey’s test. All statistical analyses were carried out using Sigmaplot 11 (Systat Software, San Jose, CA, USA). Data were regarded significantly different at P<0.05.

## Results

### Repetitive 6-Hz corneal stimulation is characterized by an increase in seizure severity and a reduction of seizure duration

We found that mice stimulated at 32 mA initially displayed a brief period(~ 10 s) of abnormal movements, including convulsions, but also some minimal stereotypies, such as head nodding and forelimb clonus, often followed by a long period(~ 100 s) of stunned posture.

**Fig 1:**
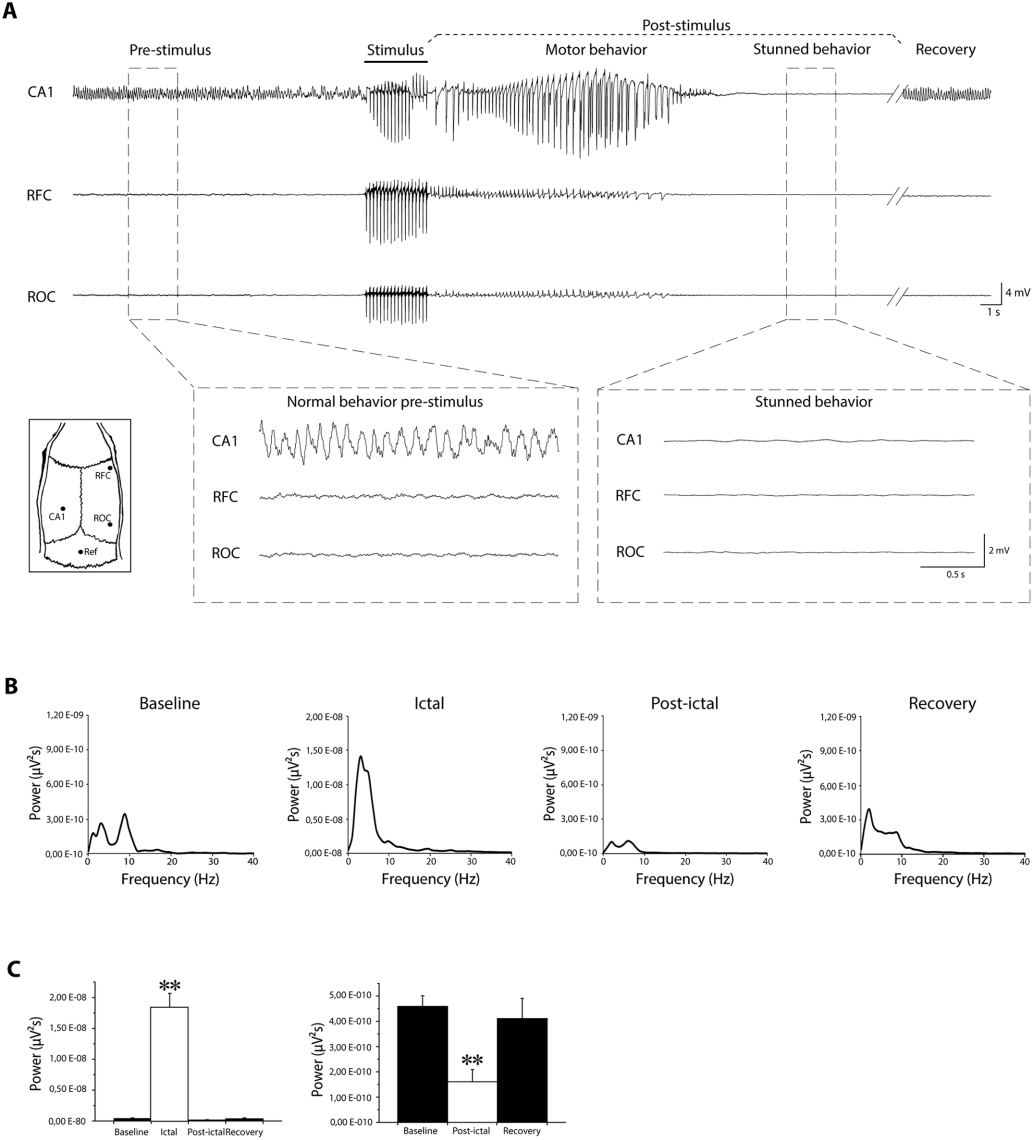
Video-electroencephalographic (EEG) recordings before and after 6-Hz corneal stimulation in mice. **(A)** EEG traces obtained from frontal and occipital cortices, and hippocampal CA1 area before, during and after a corneal stimulation from one representative mouse. Pre-stimulus represents baseline activity. Black bar indicates stimulus artifact. In post-stimulation period, two major components are evident: ictal event followed by post-ictal depression characterized by flattening of basal activity (compare traces during post-ictal depression and baseline provided at higher magnification in insets). Small inset on the left shows electrodes position. **(B)** Mean power spectra obtained by fast Fourier transformation of traces recorded by epidural electrode in frontal cortex, during baseline (n = 6), ictal (n = 6), post-ictal (n = 6) and recovery (n = 5) phases. **(C)** Histogram on the left shows that power peak during ictal event is significantly greater that power peak during baseline, post-ictal and recovery phase (** = P<0.005; Mann-Whitney rank sum test). Histogram on the right shows that power peak during post-ictal depression is significantly lower than power peak during baseline and recovery (P<0.005; Mann-Whitney rank sum test). CA1: Cornu Ammonis 1 (depth electrode); Ref: reference electrode; RFC: right frontal cortex (epidural electrode); ROC: right occipital cortex (epidural electrode).

EEG recordings (Fig 1A) revealed that the corneal stimulation evoked an ictal event, characterized by epileptiform discharges and corresponding to motor behavior, followed by a flattening of the electrical activity (Fig 1A, right inset), resembling the post-ictal depression [19] and corresponding to the stunned posture (Fig 1A). Basal activity reappeared when the animal resumed normal behavior (recovery in Fig 1A). Fast Fourier transformation of EEG traces recorded by epidural electrode implanted in frontal cortex confirmed that power spectrum of post-ictal depression was characterized by a peak, within low frequency range (Fig 1B), significantly smaller than the power peak obtained for ictal events (P<0.005, Mann-Whitney rank sum test; Fig 1C left panel), and for baseline activity (P<0.005; Fig 1C right panel).

**Fig 2:**
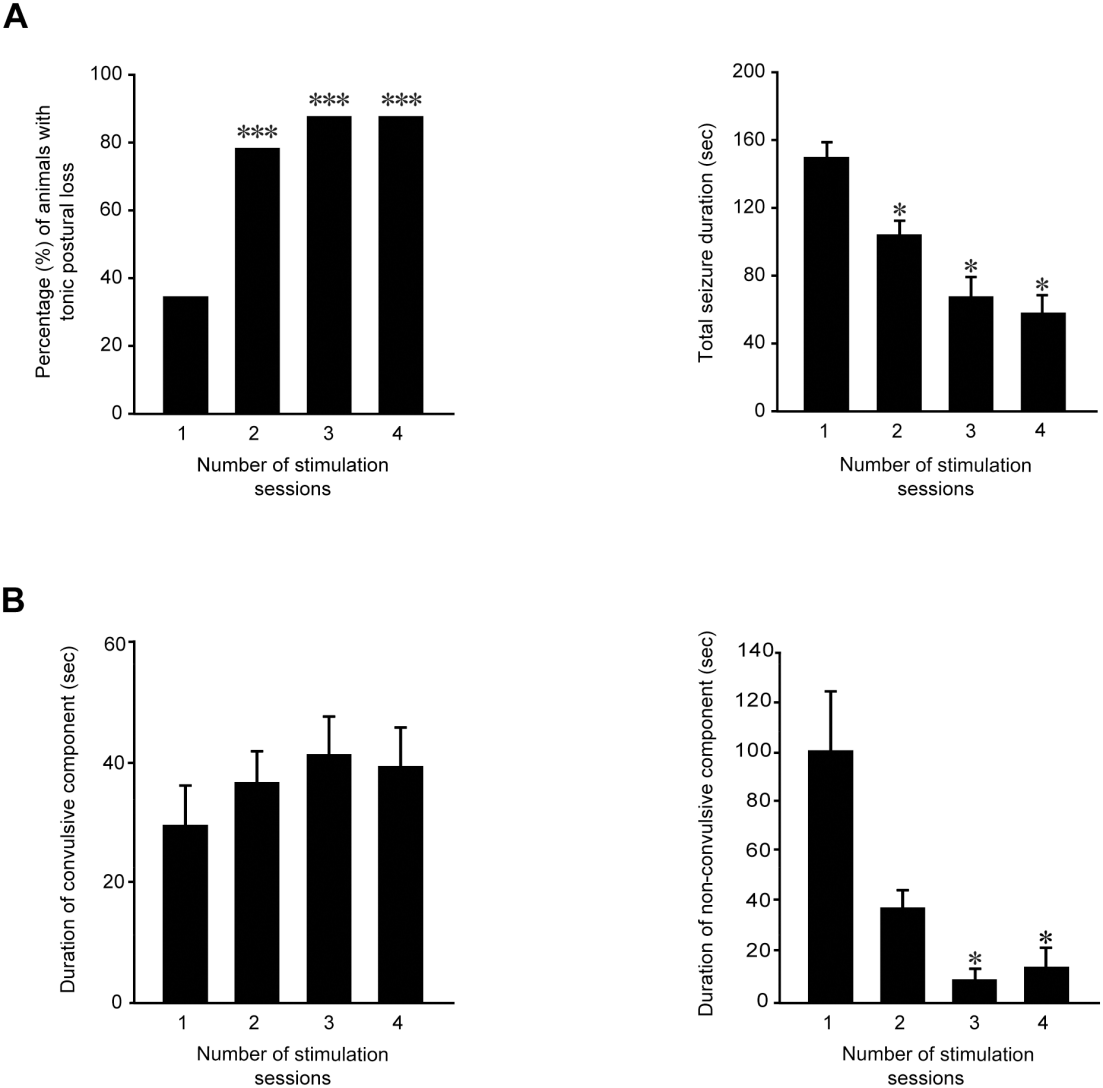
Behavioral changes in seizure severity and duration during repeated 6-Hz stimulation. **(A)** Left panel: postural loss significantly increased during repetitive stimulations (*** = P<0.001, Chi-square test). Right panel: total seizure duration decreased progressively after each stimulus session (* = P<0.05 for sessions 2, 3 and 4 vs. session 1, Dunn's test). Session 1: n = 32; session 2: n = 24; session 3: n = 16; session 4: n = 8. **(B)** Left panel: convulsive seizure duration did not change after repeated stimulations. Right panel: the reduction of total seizure duration was due to a significant decrease in duration of non-convulsive component in the animals repeatedly stimulated (P<0.05 vs. session 1, Tukey’s test). N = 8 for all experimental groups.

When mice received further stimulations, the seizure severity was significantly increased (Fig 2A, left panel; P<0.001, Chi-square test), as indicated by the higher percentage of mice displaying loss of posture during convulsions (79.17% in the second session compared with 34.38% in the first one). In spite of the more severe convulsions, the total seizure duration was significantly reduced after the first session (Fig 2A, right panel; P<0.001, Kruskal-Wallis one-way ANOVA on ranks). In particular, seizure duration observed in mice stimulated twice, thrice and four times was significantly shorter (P<0.05, Dunn's test) than in mice receiving only one stimulation. Surprisingly, we observed that duration of convulsions remained constant during the various sessions (Fig 2B, left panel; Friedman repeated measures ANOVA on ranks). On the contrary, a significant decrease in non-convulsive period was found (Fig 2B, right panel; P = 0.003, Friedman repeated measures ANOVA on ranks). In particular, groups were statistically different when comparing the first with the third and fourth sessions of stimulation (P<0.05, Tukey’s test; Fig 2B, right panel).

**Fig 3:**
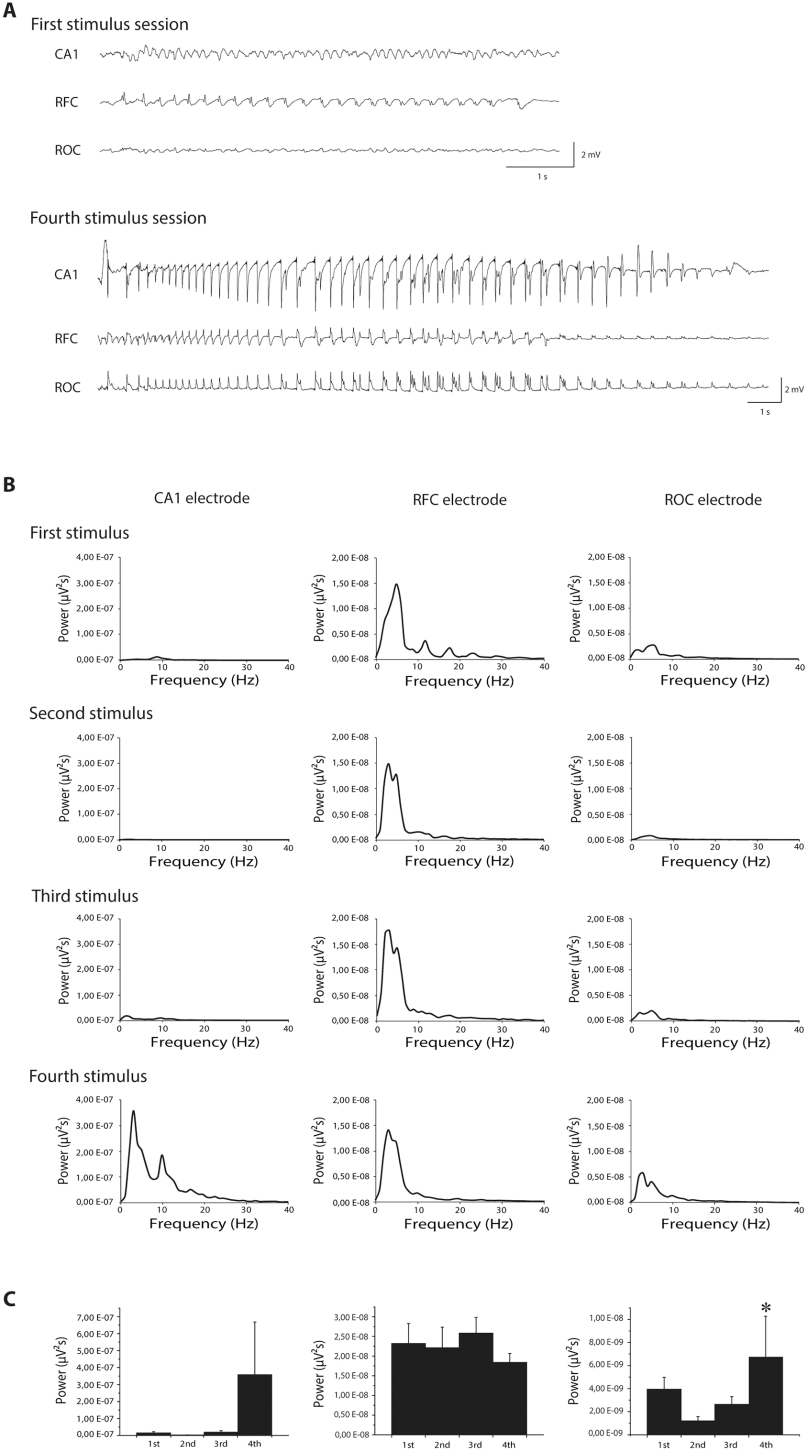
Modifications of ictal event induced by single or repeated stimulations. **(A)** Electroencephalographic (EEG) traces showing ictal events after first corneal stimulation (upper traces) and after fourth stimulation (lower traces) in one representative mouse. Note that epileptiform activity is confined in the fontal cortex after the first stimulus and spreads to occipital cortex and hippocampus after four stimulations. **(B)** Mean power spectrum of ictal events recorded by depth electrode in CA1 and by epidural electrodes in frontal and occipital cortices, induced by up to 4 corneal stimulations. **(C)** Histograms illustrating power peaks show that ictal events do not change in frontal cortex after repeated stimulations (in each session n = 5 or 6). However, power peak of ictal events progressively increases going from first to fourth stimulation in CA1 (n = 2 or 3 for all sessions, not enough to reach statistical significance) and in occipital cortex (* = P<0.05, Friedman repeated measures analysis of variance; n = 4 or 5 for all sessions). CA1: Cornu Ammonis 1 (depth electrode); RFC: right frontal cortex; ROC: right occipital cortex.

We performed EEG recordings from frontal and occipital cortices and from the hippocampal CA1 region, and we found that following the first stimulation an epileptiform activity could be consistently detected in the frontal cortex, but never in hippocampus and only sometimes in occipital cortex (Fig 3A, upper traces). This result was confirmed for the second and third stimulation. Interestingly, on the fourth stimulation epileptiform activity consistently involved the occipital cortex and, in 67% of cases, also the hippocampus, in addition to the frontal cortex (Fig 3A, lower traces). Going from first to fourth stimulation, power spectrum analysis confirmed that power peak did not change for ictal event detected by frontal cortex electrode (Fig 3B and 3C, middle panels), while it changed for ictal activity detected by occipital cortex electrode (P<0.05, Friedman repeated measures ANOVA, Fig 3B and 3C right panels) and by intra-hippocampal electrode (although in this latter case statistical significance has not been reached most likely due to a low number of cases; Fig 3B and 3C left panels).

### Repetitive 6-Hz corneal stimulation did not cause neuronal lesions or inflammation

**Fig 4:**
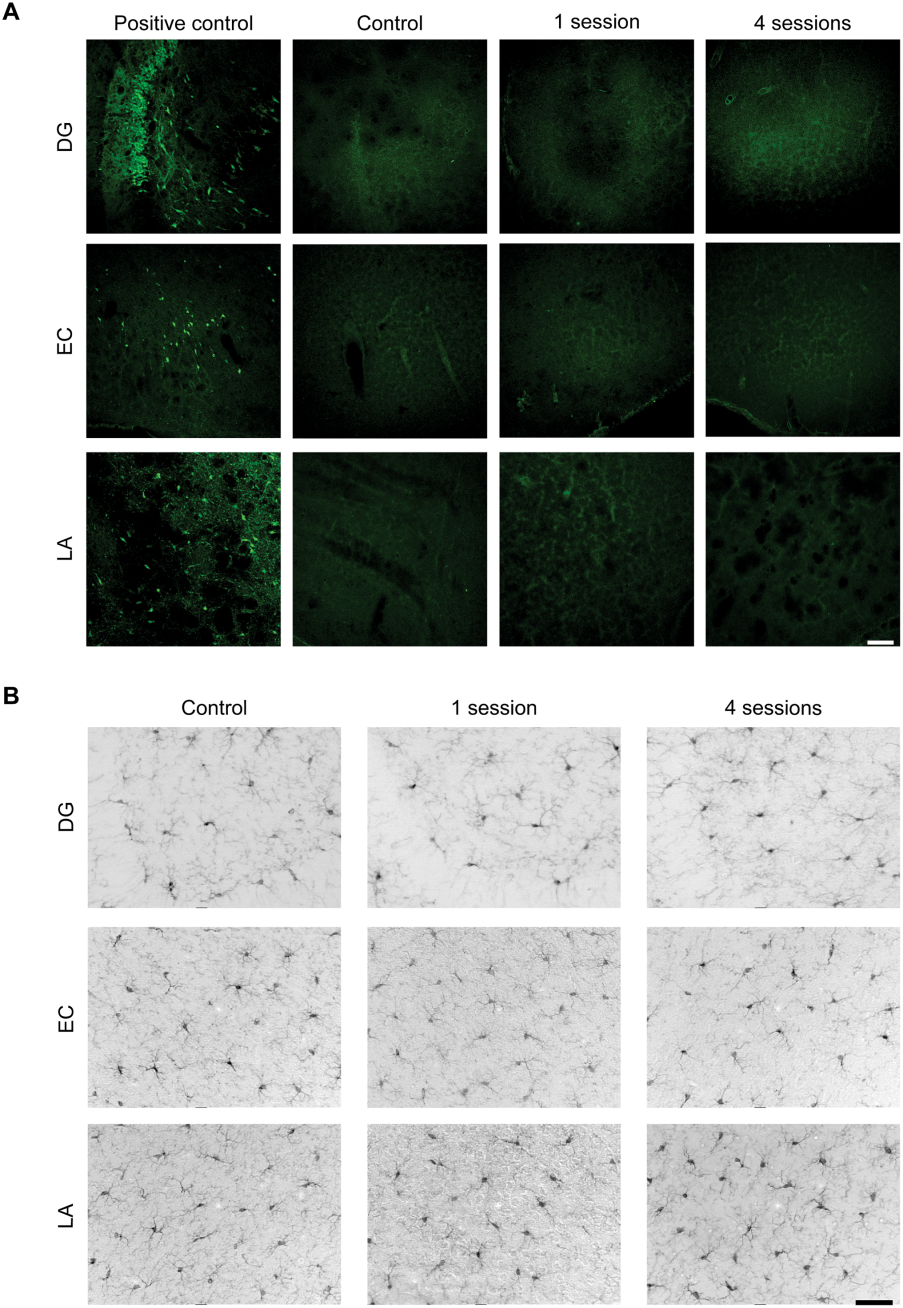
Repeated 6-Hz stimulations do not induce neuronal cell death or microglia activation. **(A)** Representative confocal images of Fluoro-Jade B positive neurons in dentate gyrus (DG), entorhinal cortex (EC) and lateral amygdala (LA) from an unstimulated mouse (control), a mouse stimulated once, and a mouse stimulated four times. On the left, same areas obtained from a pilocarpine-treated rat are used as positive control for detection of degenerated neurons [18]. This indicates that single or repeated 6-Hz corneal stimulation does not induce neuronal cell death in the analyzed brain regions. Scale bar = 100 μm. **(B)** Representative horizontal brain sections showing immunohistochemical staining against Iba1. In an unstimulated mouse (control), microglia display a typical ramified morphology in DG, EC and LA. The morphology of microglia remained similar in mice stimulated once (second column) or four times (third column) in all analyzed regions. Scale bar = 50 μm. DG: dentate gyrus; EC: entorhinal cortex; LA: lateral amygdala.

We also evaluated the possibility that occurrence of neuronal cell death and inflammation could occur in consequence of the repeated electroshocks. For this purpose, we used a fluorochrome (Fluoro-Jade B) that we previously found to clearly identify damaged neurons in the brain [14,18] and that, consistently, identified damaged neurons in sections from pilocarpine-treated rats from a previous experiment (Fig 4A, positive control; [18]). However, Fluoro-Jade B-positive neurons were not observed in DG, EC and LA or in other brain regions neither in control, unstimulated animals nor in stimulated mice, independently of the stimulation sessions (Fig 4A). Consistently with this finding, no morphological changes indicative of microglial activation were observed by Iba-1 immunohistochemistry in any considered brain region (Fig 4B).

### FosB/ΔFosB immunoreactivity was transiently increased in hippocampus and parahippocampal cortices by 6-Hz corneal stimulation

**Fig 5:**
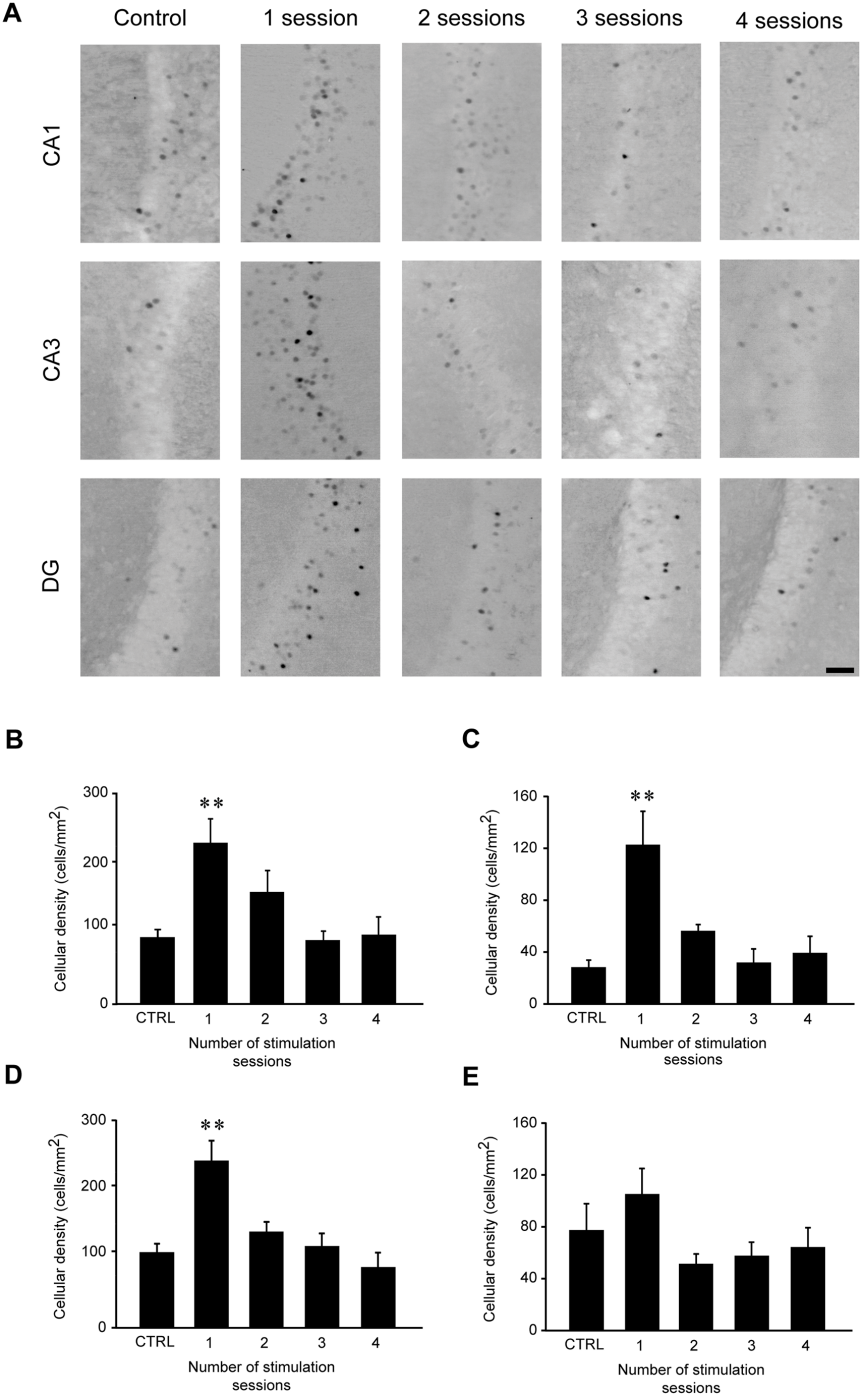
FosB/ΔFosB immunoreactivity transiently increases in CA1, CA3 and dentate gyrus (DG), but not in subiculum (Sub), after the first 6-Hz stimulation but not after repeated stimulus sessions. **(A)** Photomicrographs illustrating FosB/ΔFosB immunoreactivity in CA1, CA3 and DG regions of a representative mouse during the various stimulation sessions. Scale bar = 50 μm. In histograms, mean cell density of FosB/ΔFosB-expressing cells for the CA1 **(B)**, CA3 **(C),** DG **(D)**, and Sub **(E)** are depicted. The number of immunopositive cells was significantly enhanced after the first corneal stimulation (** = P<0.01, vs. control, Tukey’s test) in all regions but Sub, and went back to control levels after repeated stimulations. Control (CTRL): n = 5; session 1: n = 6; session 2: n = 7; session 3: n = 7; session 4: n = 6 for all regions.

**Fig 6:**
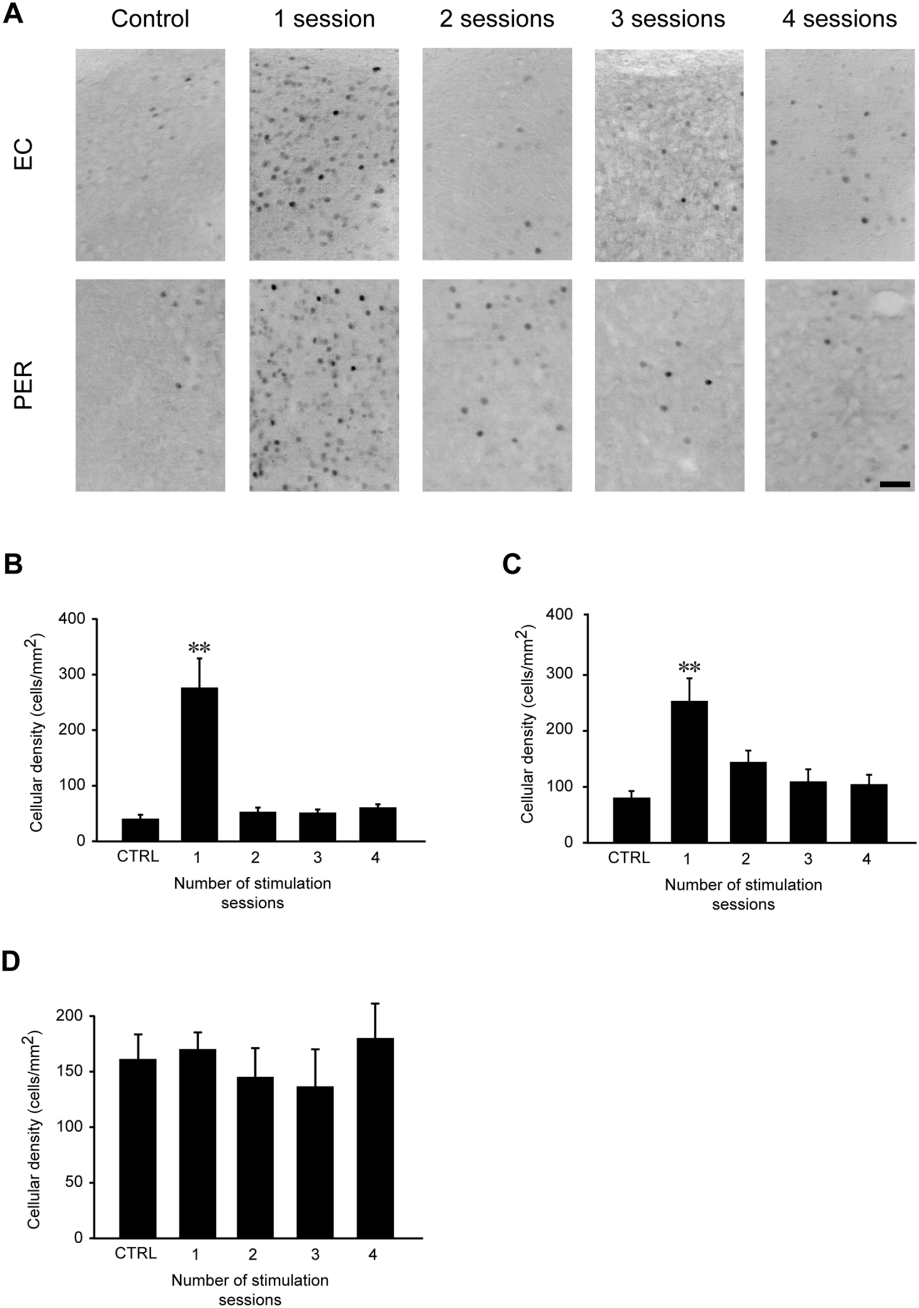
FosB/ΔFosB immunoreactivity transiently increases in entorhinal (EC) and perirhinal (PER), but not in piriform (PIR) cortex, after 6-Hz corneal stimulation. **(A)** Photomicrographs illustrating FosB/ΔFosB immunoreactivity in the EC and PER in a representative unstimulated mouse (control), and in a mouse stimulated once or 4 times. Scale bar = 50 μm. Histograms illustrating the mean cell density of FosB/ΔFosB reactivity for the EC **(B),** PER **(C)**, and PIR **(D)** are depicted. While no changes have been observed in PIR, the number of immunopositive cells was significantly enhanced after the first corneal stimulation (** = P<0.01 vs. control, Tukey’s test) in EC and PER. After repeated stimulations, FosB/ΔFosB reactivity levels went back to control values. Control (CTRL): n = 5; session 1: n = 6; session 2: n = 7; session 3: n = 7; session 4: n = 6 for all regions.

In order to determine which brain structures were activated by repeated 6-Hz corneal stimulation, we evaluated FosB/ΔFosB expression in hippocampal (CA1, CA3, DG, Sub) and cortical (EC, PER, PIR) regions. FosB/ΔFosB immunoreactivity was barely detectable in unstimulated control mice. Although no significant changes were found in Sub and PIR, in all regions of the hippocampus (Fig 5A–5D) and in parahippocampal cortices (Fig 6A–6C) a significant, bilateral increase in FosB/ΔFosB expression was measured after the first 6-Hz stimulation (P<0.01 for CA1, CA3, DG, EC and PER; Tukey’s test). However, FosB/ΔFosB expression returned to control levels when animals received further stimulations (Figs 5–6).

### Repeated 6-Hz corneal stimulation steadily increased FosB/ΔFosB immunoreactivity in LA

**Fig 7:**
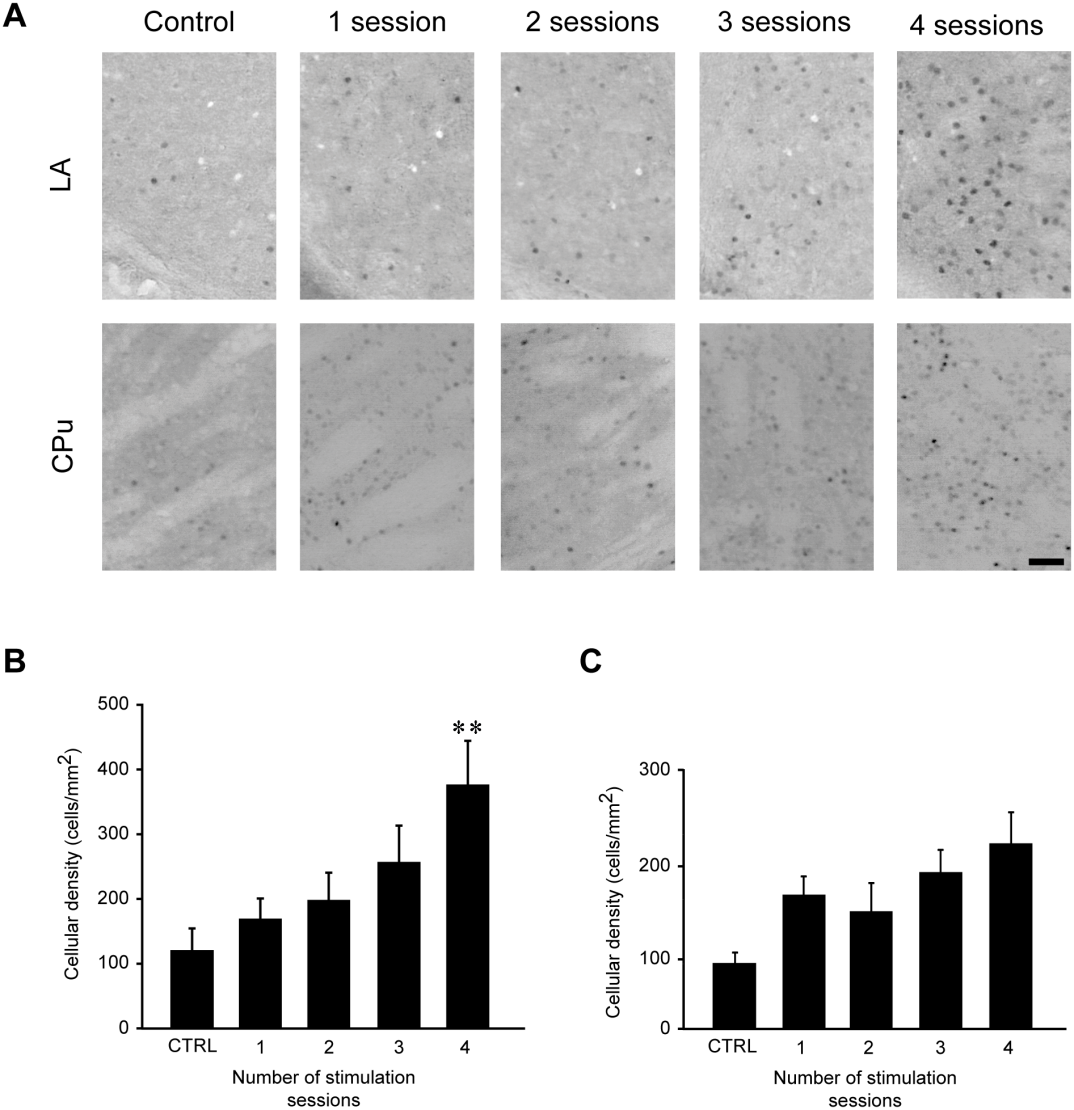
FosB/ΔFosB immunoreactivity levels in lateral amygdala (LA) and caudatus-putamen (CPu) during repeated 6-Hz corneal stimulations. **(A)** Photomicrographs illustrating FosB/ΔFosB immunoreactivity in LA and CPu in a representative mouse during the various stimulation sessions. Scale bar = 50 μm. Histograms illustrating mean cell density of FosB/ΔFosB reactivity for LA **(B)** and CPu **(C)** are depicted. The number of immunopositive cells in LA was significantly enhanced in mice receiving four stimulations compared with control group (**P<0.01, Tukey’s test). Although not significant, a trend toward increase in cell density in CPu was evident when comparing the different stimulation sessions vs. basal levels. Control (CTRL): n = 5; session 1: n = 7; session 2: n = 5; session 3: n = 5; session 4: n = 5 for LA. Control: n = 5; session 1: n = 6; session 2: n = 6; session 3: n = 6; session 4: n = 5 for CPu. CPu: caudate-putamen; LA: lateral amygdala.

Interestingly, repetitive 6-Hz corneal stimulation resulted in a progressive increase of FosB/ΔFosB immunoreactivity in LA (Fig 7A and 7B). Indeed, mice that received a fourth stimulation had a significant higher (P<0.01, Tukey’s test) FosB/ΔFosB expression than mice which were stimulated only once, and this increase in FosB/ΔFosB expression was at its highest in mice that were stimulated four times. A trend toward a progressive increase in FosB/ΔFosB expression was also observed in the CPu during repetitive 6-Hz stimulation, but the increase in FosB/ΔFosB levels was not substantial enough to reach statistical significance (Fig 7C).

### FosB induction was primarily localized in pyramidal neurons

**Fig 8:**
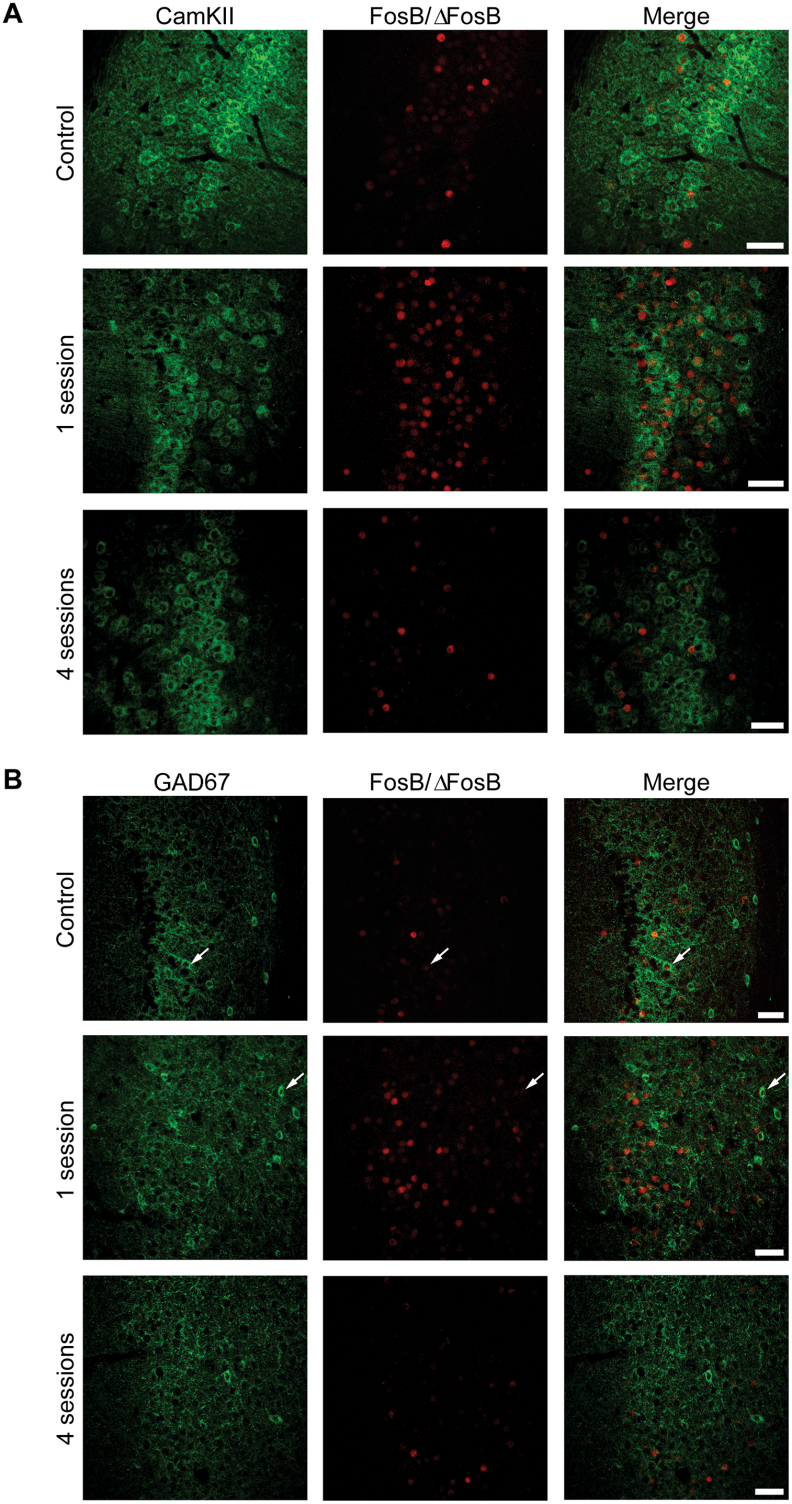
The majority of FosB/ΔFosB-immunopositive cells in the hippocampus are principal cells. **(A)** Photomicrographs illustrating double immunofluorescence experiments in the CA1 region against CamKII (green) and FosB/ΔFosB (red) in a representative mouse. The vast majority of FosB/ΔFosB-positive cells were also positive for CamKII. **(B)** Photomicrographs illustrating double immunofluorescence experiments in the CA1 region against GAD67 (green) and FosB/ΔFosB (red) in a representative mouse. Few (arrows) FosB/ΔFosB-positive cells were also positive for GAD67. Scale bars = 50 μm.

**Fig 9:**
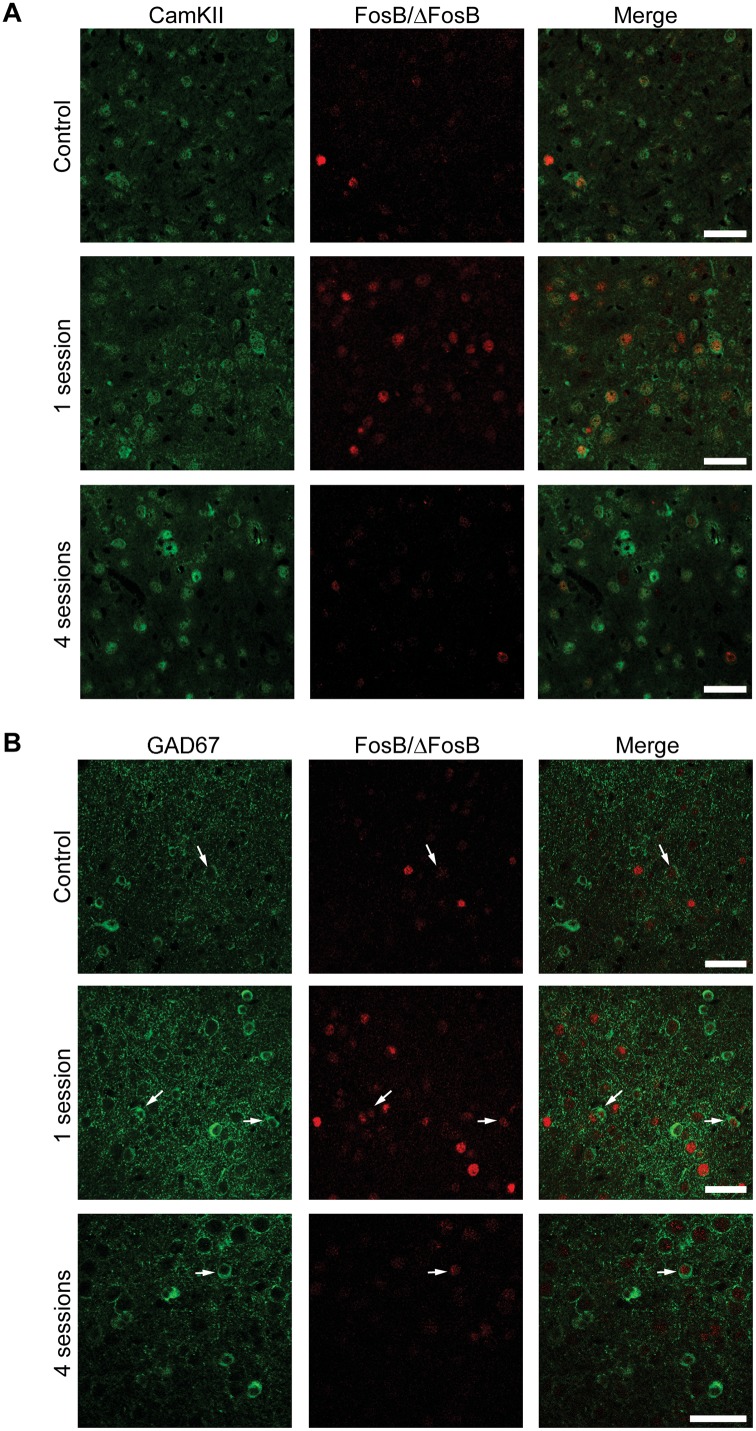
The majority of FosB/ΔFosB-positive cells in para-hippocampal cortices are principal cells. **(A)** Photomicrographs illustrating double immunofluorescence experiments in the EC against CamKII (green) and FosB/ΔFosB (red) in a representative mouse. The vast majority of FosB/ΔFosB-positive cells were also positive for CamKII. **(B)** Photomicrographs illustrating double immunofluorescence experiments in EC against GAD67 (green) and FosB/ΔFosB (red) in a representative mouse. Few (arrows) FosB/ΔFosB-positive cells were also positive for GAD67. Scale bars = 50 μm.

**Fig 10:**
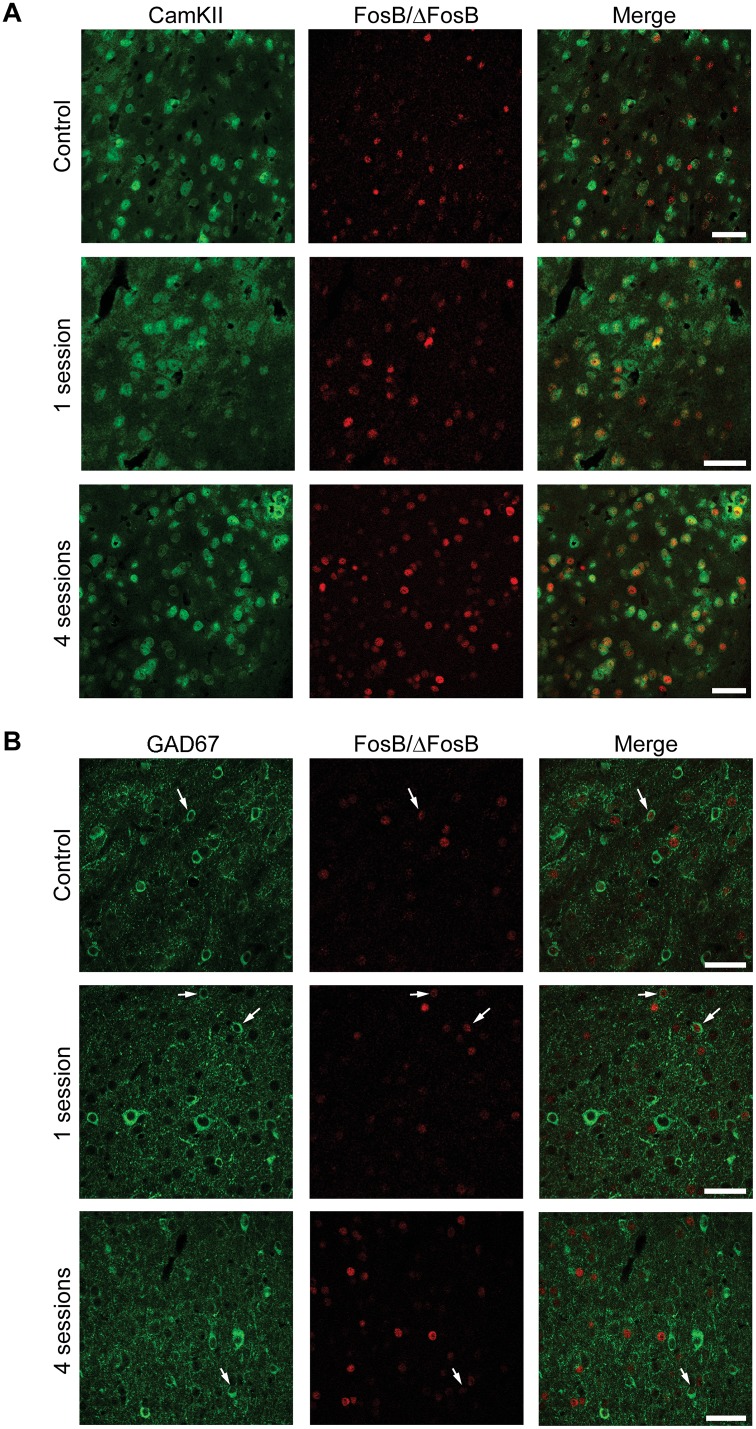
The majority of FosB/ΔFosB-positive cells in LA are principal cells. **(A)** Photomicrographs illustrating double immunofluorescence experiments in LA against CamKII (green) and FosB/ΔFosB (red) in a representative mouse. The vast majority of FosB/ΔFosB-positive cells were also positive for CamKII. **(B)** Photomicrographs illustrating double immunofluorescence experiments in LA against GAD67 (green) and FosB/ΔFosB (red) in a representative mouse. Few (arrows) FosB/ΔFosB-positive cells were also positive for GAD67. Scale bars = 50 μm.

We evaluated the phenotype of FosB/ΔFosB-expressing neurons by labelling them against CaMKII, a marker of glutamatergic pyramidal projection neurons [20] and GAD67, a marker of γ-aminobutyric acid (GABA)ergic neurons [21,22]. The vast majority of FosB-immunopositive cells were positive for CaMKII in hippocampus (CA1, Fig 8A; 77.47±2.55, % of all FosB/ΔFosB-immunopositive cells), parahippocampal cortices (EC, Fig 9A; 78.51±1.50) and extra-hippocampal regions (LA, Fig 10A; 80.65±1.60). Most importantly, no differences in co-localization were observed between experimental groups (control: 77.99±1.27; 1 session: 78.89±1.40; 4 sessions: 80.99±2.19; one-way ANOVA). These findings suggest that 6-Hz stimulation primarily activates pyramidal neurons. Accordingly, some FosB/ΔFosB-positive cells were also interneurons, being positive for GAD67 (CA1, Fig 8B, 14.22±0.71; EC, Fig 9B, 16.76±2.33; LA, Fig 10B, 19.05±3.81). Again, no differences in co-localization were observed among experimental groups (control: 14.61±1.80; 1 session: 15.76±1.75; 4 sessions: 16.89±1.41).

## Discussion

We have evaluated a modified version of the 6-Hz corneal stimulation test in order to analyze: (i) the possible changes in seizure phenotype in response to repeated stimulation, (ii) the degree of neuronal network activity following the repeated stimulation. First, in spite of the initially observed mild, psychomotor seizures, we found that more severe tonic-clonic convulsions rapidly developed by subsequent 6-Hz corneal stimulations. Second, seizure duration paradoxically decreased because of a more rapid recovery from the period of stunned posture that followed convulsions. Third, by using video-EEG recordings we found that ictal activity was not present in the hippocampus until the last corneal stimulation was applied. Fourth, by imaging neuronal activity with immunohistochemistry, we identified the LA as the region in which FosB/ΔFosB immunoreactivity was steadily increased in response to the repeated stimulations. Overall, the changes found in the amygdala and the associated appearance of epileptic activity in the hippocampus are compatible with the occurrence of an epileptogenic process in the limbic system.

In agreement with the observations made by others [8,9,23], we found that mice repeatedly stimulated at 32 mA initially displayed minimal abnormal movements (psychomotor seizures), and a long period of immobility (stunned posture). These changes were not previously defined by video-EEG recordings in 6-Hz stimulated mice, as we instead did in our modified version of the model. As expected, the video-EEG recordings revealed a strict correspondence between the motor behavior elicited by 6-Hz electroshocks and ictal activation on the EEG; moreover, this relationship was maintained during the various sessions of corneal stimulation. We also observed that the stunned behavior that followed the motor responses, and that preceded the recovery to normality, corresponded to flattening of the EEG, a phenomenon which has been largely addressed but partially clarified [24–26]. Notably, motor responses became more severe, but the overall seizure duration decreased immediately after the second session of corneal stimulation, which is to our knowledge an unreported finding. These session-dependent changes did not further progress by the fourth stimulation. At variance, on the fourth session we noticed that EEG seizures generalized to the hippocampus.

The behavioral changes we observed in this modified 6-Hz corneal stimulation model are complex and have probably different explanations. First, the appearance of more severe convulsions recalls the progressive aggravation of seizures induced by kindling protocols [27], including the so-called “corneal kindling” at 50-Hz/60-Hz [28–31], but we neither used subthreshold stimuli nor applied frequent stimulation cycles. Second, the reduction in seizure duration, instead, could be related to appearance of resistance to seizure induction as described in rats previously subjected to electrical stimulation and subsequently treated with a chemoconvulsant, specifically in models of TLE [32,33]. However, the changes observed in our model specifically regarded the time interval enclosed between termination of convulsions and recovery of normal behavior. To this regard, it has been suggested that seizures are terminated by the activity of inhibitory mechanisms that, on the other hand, may outlast the motor response and could be responsible for the phenomena encountered in the postictal period, including behavioral depression [26,34–36]. Notably, loss in efficiency of inhibitory mechanisms could be responsible for both the increased seizure severity and the reduction in stunned posture observed in our model.

The most interesting result of our study was the late appearance of ictal activity in limbic structures, as shown by the CA1 electrode. This event was temporally unrelated to the other described changes in seizure characteristics and, for this reason, probably relied on different mechanisms. Using FosB/ΔFosB immunoreactivity as a marker of neuronal activation, we also found that FosB/ΔFosB levels became particularly pronounced in the LA at the same time interval in which ictal activity appeared in EEG recordings from CA1. The LA is heavily connected with the basal amygdala [37], which is directly connected to CA1 through the caudomedial portion of its parvicellular division [38]. Although we had not the possibility to evaluate FosB/ΔFosB levels in the basal amygdala of horizontal brain sections, in few coronal sections of 6-Hz corneally stimulated mice we noticed a widespread immunopositivity in this region (data not shown). The amygdala projection to CA1 was suggested to be involved in the seizure spread to the hippocampus in kainate-treated rats [39], and it could have played a role in the appearance of hippocampal ictal activity in the modified 6-Hz corneal stimulation model.

A progressive accumulation of *fosB* related products have also been described for other chronic treatments, by which FosB/ΔFosB proteins were shown to increase their levels in relation with repetition of the stimulus [40]. As different factors are able to induce *fosB* expression [40], the observed changes in FosB/ΔFosB immunoreactivity may not be related to epileptic activation of neuronal networks; however, changes similar to those observed in the 6-Hz corneal stimulation paradigm have been found in several models of epilepsy [10–15] and after exposure to electroconvulsive seizures [41]. If induction of FosB/ΔFosB immunoreactivity in LA depended, at least in part, on seizure induction, the possible consequences for seizure propagation in the limbic system could be very important in view of the role played by the amygdala in TLE [42]. Indeed, the timing of FosB/ΔFosB induction in LA, and the appearance of ictal activity in the CA1 hippocampal region, were both very suggestive for a possible relationship between the two phenomena. On the other hand, the changes found in FosB/ΔFosB expression in the other brain regions seemed to be related more to the initial exposure to corneal stimulation rather than to the progression in seizure severity observed by repeating the test, suggesting a novelty effect then followed by adaptation as noticed in another, different model [43].

## Conclusions

Our investigation provides a thorough analysis on the cerebral regions involved in seizures evoked by 6-Hz corneal stimulation. In particular, by providing evidence that exposure to repeated stimulations changed the patterns of neuronal activation, our experiments identified a role for the amygdala in the generalization of seizures to the hippocampus, a phenomenon described in TLE, one of the major type of refractory epilepsy. This modified paradigm of 6-Hz corneal stimulation may prove to be useful for studies aimed at disclosing mechanisms involved in epileptogenesis, as well as to evaluate the antiepileptogenic potential of drugs or other, alternative treatments which require a prescreening in a simple, easy-to-use animal model.
